# Adherence and Concordance of Influenza and Pertussis Vaccination Coverage in Pregnant Women in Spain

**DOI:** 10.3390/ijerph16040543

**Published:** 2019-02-14

**Authors:** Noelia Rodríguez-Blanco, José Tuells, Rafael Vila-Candel, Andreu Nolasco

**Affiliations:** 1 Department of Obstetrics and Gynaecology, Hospital Universitario del Vinalopó, Spain C/Tonico Sansano Mora, 14, 03293 Elche, Spain; nrodriguez@vinaloposalud.com; 2 Department of Nursing Universidad CEU Cardenal Herrera, Plaza Reyes Católicos, 19, 03204 Elche, Spain; 3 Cátedra Balmis Vacunología, University of Alicante, Campus de San Vicente Raspeig, Ap.99, E-03080 Alicante, Spain; 4 Department of Obstetrics and Gynaecology, Hospital Universitario de la Ribera, FISABIO, Spain, Crta. Corbera km 1, 46600 Valencia, Spain; vila_rafcan@gva.es; 5 Unidad Mixta de Investigación para el Análisis de las Desigualdades en Salud y la Mortalidad FISABIO-UA, Departament of Community Health, Preventive Medicine and Public Health, and Science’s History, University of Alicante, Carretera de San Vicente del Raspeig s/n, 03690 Alicante, Spain; nolasco@ua.es

**Keywords:** influenza, pregnancy, immunisation, vaccine coverage, pertussis vaccine, midwife, nominal records

## Abstract

*Background*: Pregnant women should receive two vaccines during pregnancy due to maternal-foetal complications and risks as well as the influenza and pertussis vaccinations. The goal was to evaluate vaccination coverage against influenza and pertussis in pregnant women, following midwife professional advice during the pregnancy follow-up; *Methods*: Prospective cohort study of 1017 pregnancies during the vaccination campaign in 2015–2016. To estimate the degree of consistency between the coverage declared by mothers and that registered in the Nominal Vaccination Registry (NVR), we used the Cohen’s kappa index (k); *Results*: 95.4% were registered in the NVR. Vaccination coverage recorded against influenza was 64.2% (95% CI: 61.2–67.2), and 89.8% (95% CI: 87.9–91.7) against pertussis. The coverage of the pregnant women interviewed was 61.7% (95% CI: 58.1–67.3)) for influenza and 92.3% (95% CI: 91.4–95.3) for pertussis. Subsequent interviews of 67.2% of the women produced a kappa agreement index between the data obtained from interviews and those recorded in the NVR of 0.974 (IC95%: 98.0–99.6) for influenza, and 0.921 (IC95%: 98.1–99.7) for pertussis. The women identified midwives as the main source of vaccination information and advice 88.4% (IC95%: 85.8–90.9); *Conclusions*: The NVR is an effective platform for estimating immunisation coverage in pregnant women. The degree of agreement between declared vaccinations and registered vaccinations was high for both vaccines.

## 1. Introduction

A current priority for health authorities is that of achieving adequate immunisation coverage of the whole population and especially risk groups, including pregnant women [1]. Vaccination against influenza has been recommended in Spain since 2005 and since 2014 against pertussis, being the only two vaccines administered during the pregnancy control program [2,3].

Pregnant women sometimes develop complications due to seasonal influenza associated with higher maternal morbidity and mortality, which lead to increased hospital admissions and poor perinatal outcomes [1,2,4].

The influenza vaccination has been seen to be associated with a lower risk of foetal death (RR 0.73; (95% CI: 0.55 to 0.96)) [5], in addition to decreasing the risk of prematurity due to childbirth preterm and the newborn being small for his gestational age [6,7].

For this reason, several international organizations recommend administering the influenza vaccine in any trimester of the pregnancy [8,9]. Although the recommendation is backed by substantial evidence and the safety of the vaccine is guaranteed, vaccination coverage against influenza and acceptance of immunisation during pregnancy is suboptimal [10]. Spanish vaccination records are diverse but they all reveal low vaccination coverage, never exceeding 40% [11,12]. Worldwide, the influenza vaccination coverage is irregular, ranging from 15–43% in Europe [13] and reaching 50% in the USA [14].

Vaccination against *Bordetella pertussis* is also recommended for all pregnant women due to cyclic pertussis outbreaks [15,16]. Since 2011, the number of pertussis cases has increased across different regions of the world, including the European Union, in children and adolescents, as well as adults [17]. In Spain, the incidence rate in 2011 was 6.73 per 100,000 inhabitants/year, though it reached 523 per 100,000 inhabitants/year in infants from 0 to 2 months in 2014, a high incidence for this age group, who may not get the vaccine until they are two months of age [18]. In Spain, health competencies are decentralised and are attributed to Autonomous Region Administrations. Spain’s 17 Autonomous Communities (ACs) are independent in health matters and each AC adapts its health resources according to the needs of its population [19]. Between 2014 and 2016, all ACs decided to vaccinate their pregnant population [2,3].

In Spain, the first time that coverage of the pertussis vaccine in pregnant women was published was in 2018, according to the data published by the Ministry of Health and Social Services of Health (MSCBS) [20]. According to these data, the coverage was high, reaching 82.7% and 89.2% in the Valencian Community (VC), and the program has had a substantial impact to the extent that the incidence of pertussis has declined in all age groups, especially in infants under three months (17.99/100,000 to 8.80/100,000 inhabitants/year) [20]. In the same period, coverage reached for the influenza vaccine in pregnant women throughout the country was 27.6%, although the VC obtained a coverage greater than 43.7% [21].

The Nominal Vaccination Registry (NVR) portal belongs to the Government of the VC. It keeps electronic records of the vaccination status of all people born after 1994 [22]. Health professionals that are part of the immunisation program are required to use the NVR and enter a record of each vaccine administered to any child or adult in the electronic registry. Each AC collects and forwards the information of its nominal or numerical registers to the MSCBS enabling to calculate the total number of vaccinated people in the country. An individual’s vaccination history must contain vaccines administered at all stages of life: early childhood, at school, gestational age, working age, etc. These records are useful to know an individual’s history of vaccination, population coverage, to detect unvaccinated groups and to evaluate the impact of the immunisation program [23]. The records are fed with demographic data on the population susceptible to be vaccinated (denominator) and with the sum of vaccinated people (numerator), leading to the calculation of vaccination coverage (VC = N/D). A registry should have the following characteristics: flexibility, accessibility, security and confidentiality of the data. The active role that health professionals can play has been demonstrated: they provide proper immunisation advice, which reduces rejection due to ignorance, fear or the belief that the vaccine is dispensable [24,25,26]. Midwives also contribute to dissipating doubts on vaccination during their follow-up of pregnant mothers [27]. Although vaccination during pregnancy is a relevant public health issue, few studies have explored vaccine coverage by jointly examining two sources of information: vaccination records and statements by pregnant women themselves. We aimed to obtain the rate of recorded vaccination coverage against influenza and pertussis in a group of pregnant women who received vaccination advice from their midwives, as well as the rate of coverage declared by mothers after pregnancy, in order to then assess the level of agreement between both sources of information. 

## 2. Materials and Methods

### 2.1. Design, Population, and Sample

A prospective cohort study was conducted in two health departments of the VC (Spain) on pregnant women assigned to the pregnancy monitoring and control program.

The study was conducted in the Valencian Community in the period included the seasonal influenza vaccination campaign carried out between 15 October 2015 and 31 January 2016. The health departments of Torrevieja (TV) and Elche-Crevillente (EC) had a population of 157,000 and 146,000 inhabitants, respectively, and a yearly average of 1200 and 1500 births. 

The influenza and pertussis vaccine policy in the TV and EC health departments is modelled on the national policy, according to which vaccines are offered systematically to all women by community midwives and family doctors free of charge. In 2016 at VC, the vaccination coverage against influenza in pregnant women represented 35.0%, while the coverage for pertussis was unknown [28].

The sampling was consecutive and included all the women who followed the pregnancy control program during consultations with midwives in the two health departments during the study period. As the safety of the influenza vaccine is well established, its administration is recommended at each trimester of gestation. Women are offered immunisation on the third trimester, ideally between weeks 27 and 36 of the gestation for pertussis vaccine. Women with language difficulties, foetal death, and/or contraindications regarding vaccine administration were excluded.

A minimum necessary sample size of 549 pregnant women was calculated to enable drawing estimations of vaccine coverage proportions, with a confidence interval of 95%, 5% for accuracy, an expected coverage of 50% and a follow-up loss rate of 30%. 

Our study applied the ethical principles for medical research established in current legislation and was approved by the Research Commission of the participating departments following its authorization by the Spanish Agency for Medicine and Health Products (AEMPS to use its Spanish acronym). The study was approved by The Research Commission’s (ERC-IC) Research Ethics Committee at the Vinalopó Hospital (#134-14). Considerations such as confidentiality, voluntary participation, and full information on the nature of the study were extended to all participants. The attending midwives recruited the women after clinical consultations and obtained their informed consent to participate in the study. In this situation, there may be a fine balance between consent and coercion but, as per the protocol approved by the Research Ethics Committee, the women were reassured that their participation—or lack thereof—would have no influence on their clinical care.

### 2.2. Data Collection Tools

As a preliminary step, midwives were trained over a range of sessions on the characteristics of the study and the type of vaccine advice. This training was carried out two months before the start of the seasonal influenza vaccination campaign. All the midwives in the 10 health centres of both health departments participated. 

Once the seasonal vaccination campaign began, the midwives started including all the women who came to see them for a consultation. The procedure consisted of requesting the verbal consent of the pregnant women to participate in the study, collecting their personal data as well as explaining the importance of being vaccinated against influenza and pertussis. The information collected consisted of the sociodemographic data and obstetric characteristics of the pregnant women; health department (TV/EC); country of origin (Spain/Not Spain); average age; previous pregnancies (1, 2, ≥3); previous abortions and the number of births (0, >1). All the pregnant women agreed to participate in the study. 

#### 2.2.1. First Phase of the Study

Once the seasonal vaccination campaign was completed, the NVR records of the vaccine status against influenza and pertussis of the total number of women included in the study were checked. The first phase of the study concluded with the results of vaccine coverage according to NVR. 

#### 2.2.2. Second Phase of the Study

The second phase of the study consisted of interviewing mothers to obtain information about the vaccines they received. This information was collected through telephone interviews conducted by a trained midwife. The questions asked to the women after the delivery were the following: (1) Were you vaccinated during the pregnancy? (2) Who recommended the vaccination? (3) What vaccines have you been administered? (4) Where were you vaccinated? (5) Did you experience any side effects after the vaccination? 

The second phase of the study was concluded with the vaccination coverage described by the mothers. 

#### 2.2.3. Final Analysis of the Study

**Figure 1:**
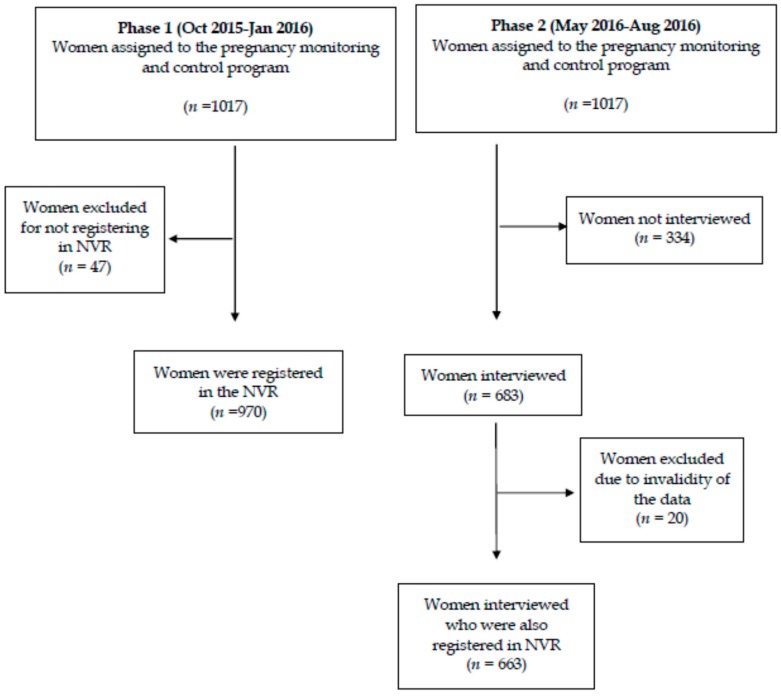
The flow chart of the study.

A final analysis compared the vaccination coverage obtained by the two data sources (the NVR registry and mothers’ declarations) to identify the degree of agreement between the two (Figure 1).

### 2.3. Methods of Analysis

Statistical analysis was carried out using the SPSS program (IBM Corp. Released 2011. IBM SPSS Statistics for Windows, Version 20.0, Armonk, NY, USA). Frequencies and percentages of the categories were calculated for all the variables, as well as 95% confidence intervals (95% CI). The standard deviation (x ± SD) of the quantitative variables mean was calculated. The Chi-square test was used to analyse the statistical significance of the differences in the percentages of vaccine coverage between the various categories. 

To evaluate the adjusted effect of the variables of age, previous parity, previous abortions, month of visit to the midwife, country of origin and health department on vaccination rejection, multivariate logistic regression models and their odds ratio (OR) were constructed, taking the non-vaccination variable as a response and the rest as explanatory variables. To assess the agreement between the two sources of information on vaccination coverage against influenza and pertussis, i.e., between mothers’ verbalization of the vaccination status and NVR records, Cohen’s Kappa index (k) and the percentage of concordance were estimated. The level of accepted statistical significance was *p* < 0.05. 

## 3. Results

### 3.1. First Phase of the Study

During the first phase of the study, a total of 1017 pregnant women agreed to participate and received vaccination advice on influenza and pertussis, provided by the midwives of the ten health centres of both departments (EC and TV). There were no follow-up losses during this phase. 

The EC department contributed the largest number of women to the study, i.e., 57.1% (581/1017). Participants of Spanish origin accounted for 69.2% (704/1017), with an average age of 30.4 ± 5.6 years. For 90.4% (920/1017) of the women, this was their second pregnancy and 93.5% (951/1017) had never had an abortion. The distribution by trimesters of pregnancy of the women included in the control program was 28.0% (284) for the first trimester, 35.8% (365) for the second trimester, and 36.2% (368) for the third trimester. November was found to be the month where the highest number of influenza vaccine recommendations had been given, amounting to 65.7% (668/1017), and the month of January registered the lowest number of vaccination recommendations, i.e., 2.6% (27/1017). 

The vaccination status was then checked in NVR records. Of the 1017 participating women, 970 (95.4%) had a registry in the NVR, representing a loss of 4.6% (47). Of these, a total of 57.7% (560/970) were administered both vaccines, while 3.7% (36/970) of the women were administered no vaccine at all during pregnancy. Vaccination coverage against influenza registered in the NVR was 64.2% (*n* = 623 (95% CI: 61.2–67.2)), and against pertussis, 89.8% (*n* = 871 (95% CI: 87.9–91.7)). A total of 6.5% (*n* = 63 (95% CI: 0.4–12.6)) of the pregnant women were vaccinated against influenza, but not pertussis.

Information on vaccine coverage of both vaccines drawn from the NVR relating to the different sociodemographic and obstetric variables are shown in Table 1. Statistically significant differences can be observed (*p* < 0.01) for the variables: department (greater pertussis vaccination in EC and greater vaccination of influenza in TV), country of origin (greater influenza vaccination in non-Spanish women and greater pertussis vaccination in Spanish women) and age (influenza vaccination in younger women and pertussis in older women).

Influenza vaccination was higher in the second pregnancy (602/920) (65.4% (95% CI: 62.3–68.5)) and was lower in women who had previously had an abortion (9/19) (47.4% (95% CI: 24.9–69.8)). No statistically significant differences were found according to the previous births and abortions in the case of the pertussis vaccination. The intention to be vaccinated of the women after the visit to the midwife meant a greater coverage (596/765) (77.9% (95% CI: 75.0–80.8)). A total of 86.8% (178/205) (95% CI: 82.2–91.4) of the women who did not want to be vaccinated were not immunised, with statistically significant differences (*p* < 0.0001).

### 3.2. Second Phase of the Study

In the second phase of the study, a telephone interview was conducted with each of the women after delivery. Of the 1017 initial participants, 334 (32.8%) were not interviewed for the following reasons: 272 (81.4%) for not being able to be reached by telephone after 2 attempts, 46 (13.8%) did not wish to respond to the survey and 16 (4.8%) for having had an abortion during the study period. The total number of women interviewed was 683 (67.2%); they were distributed by health departments in the following way: 267 (39.1%) in TV and 416 (60.9%) in EC. 

The total number of pregnant women interviewed who were also registered in the NVR during pregnancy was finally 663 (65.2%) after the elimination of 20 (2.9%) records that contained registration errors. As shown in Table 2, the coverage of the pregnant women interviewed was 61.7% (*n* = 409 (95% CI: 58.1–67.3)) for influenza and 92.3% (*n* = 612 (95% CI: 91.4–95.3)) for pertussis.

Women acknowledged having been informed by midwives in 88.4% (*n* = 604 (95% CI: 85.8–90.9)) of the cases, by the family doctor in 6.4% (*n* = 44 (95% CI: 0.8–13.6)) of the cases, by family and friends in 3.1% (*n* = 21 (95% CI: 0.0–10.5)) of the cases, by the obstetrician in 1.2% (*n* = 8 (95% CI: 0.0–8.7)) of the cases, and by an unknown source in 7.6% (*n* = 52 (IC95%: 0.4–14.8)) of the cases.

After adjusting multivariate logistic regression models, in the case of influenza, only the health department variable (higher vaccination in the TV department) was found to have a significant association (*p* < 0.01) with OR = 1.92 (95% CI: 1.39–2.67). In the case of pertussis, a significant association (*p* < 0.01) was found between non-vaccination and age (lower vaccination coverage at younger ages, OR = 0.91, 95% CI: 0.87–0.96) and the country of origin (lower vaccination in non-Spanish born women, OR = 0.41, 95% CI: 0.24–0.82). 

The analysis of concordance between the data obtained through the interviews and the information registered in the NVR showed that there was a high level of agreement. The kappa values and their concordance percentage were k = 0.974 (*p* < 0.001) and a concordance of 98.8% (CI95%: 98.0–99.6) for influenza vaccination and k = 0.921 (*p* < 0.001) and a concordance of 98.9% (CI95%: 98.1–99.7) for pertussis.

The side effects recorded for the influenza vaccine related to 8.6% (36/421) of women and were always mild. When asked about the characteristics or type of side effects experienced, the most mentioned side effect, in 47.2% (17/36) of cases, consisted in symptoms similar to those of common colds (with no fever), 33.3% (12/36) of cases manifested pain and inflammation in the arm where the vaccine had been inoculated, 16.7% (6/36) had the flu and a single case reported a hospital admission due to risk of premature birth following the vaccination. 

The side effects recorded for the pertussis vaccine were also scarce, accounting for 10.6% (67/630) of cases; all were mild. A total of 91.0% (61/67) were due to pain in the arm where the vaccine had been administered accompanied by inflammation over several days and fever in 8.9% (6/67) of the cases (Table 3).

## 4. Discussion

Vaccinations during pregnancy are an essential public health initiative [29]. These vaccinations not only protect the mother but confer immunity to the foetus during the pregnancy and to the infant for up to 6 months due to placental transfer of maternal IgG. It may also be good to mention that the immunisation of mothers will also provide protection to their offspring during breastfeeding [30]. 

The main objective pursued by any vaccination program is to achieve high vaccination coverage [31]. Some studies have evaluated both vaccination coverage against influenza and pertussis in pregnant women or established the consistency between vaccination records in the NVR and mothers’ statements regarding the vaccines they received during pregnancy [18].

In our study, a coverage of 64.2% against influenza was obtained, a much higher figure than that obtained for the rest of the Valencian Community (35.0%), or for Spain (30–40%) [2,11]. In the USA, the coverage was documented at around 50.0% following the 2009 influenza pandemic, although most publications report a lower percentage, closer to 45.0% [10,32]. Regarding the pertussis vaccine, we obtained a coverage of 89.8%, which exceeds that found in different studies in Europe, such as Belgium (39.0%) or the UK (70.0%) [13,33] and similar to that recorded in another health department of the Valencian Autonomous Community [2,11]. Both vaccines are recommended during pregnancy follow-ups and may or may not coincide over time [12], although they had varying levels of acceptance by women in our case. 

In the study by Amirthalingam et al., vaccinations against pertussis were found to have increased during the winter months, coinciding with the influenza vaccination campaign (a result similar to that found in our study), a fact that could act as a facilitator to ensure high immunisation coverage for pertussis [15]. We also observed that vaccination against influenza was greater during the first two months of the seasonal campaign, when pregnant women also received more vaccination advice, producing a downward trend, as also described in Vilca’s study [34]. This fact can be attributed to some kind of professional “wear and tear” or simply that the intensity of the seasonal influenza campaign is greater during that period. 

Intention to be vaccinated towards the influenza vaccine was high after scheduled visits to midwives, coinciding with other studies [14,35], where health professional advice has been found to be the most important predictor of proper immunisation. In Spain, pregnancy check-ups are mainly carried out by midwives and obstetricians. Several studies have found that pregnant women acknowledge midwives as their main source of vaccination advice, ahead of obstetricians and family doctors, which is compatible with our findings [2]. However, despite the information source being one and the same for influenza and pertussis, we found a notable difference (25.0%) in coverage between both vaccines.

Various factors can explain these differences. Firstly, health providers do not place an equal emphasis on the influenza vaccination than on other vaccines, as indicated by different authors [36,37]. Decisions related to vaccination may be influenced by the type of information provided, the way of communicating and the professional’s attitude regarding the vaccine [38]. The process is thus interactive and complex. In our case, the group of midwives participating in the study received specifically designed additional training sessions prior to the seasonal vaccination campaign. Differences, however, were not reduced, though better coverages than those published to date were obtained.

A second factor is the need to transmit and include the message of protecting new-borns through maternal immunisation. This message is more widely disseminated in the case of the pertussis vaccine, which usually receives greater acceptance. Health professionals should avoid highlighting the difference between influenza and pertussis vaccines when giving vaccination advice to women [39]. The last factor, though by no means the least important one, could be that women feel that the influenza vaccine is unnecessary, as indicated in other studies [13,14]. 

To know if a woman is suitably vaccinated, she must be able to give a reliable answer to three points: she must know who advised her, where she was vaccinated and confirm that she has been vaccinated [22]. These three questions were checked after the telephone interview in the postpartum period, and the two latter questions in the vaccination registry (NVR). The analysis of concordance between vaccinations declared after the interviews and those recorded in the NVR produced significant kappa index values and concordance percentages. A recent study in Spain achieved concordance indicators that were well below those obtained in the present study [40]. In our study, data loss or insufficiency in the registry was similar to that of other European registry systems [41] or was below that found in other studies [42]. 

Based on the multivariate analysis, the health department variable was found to have the greatest weight as an explanatory variable of influenza vaccination. Greater coverage was associated with the health department, and pregnant women in the Torrevieja department received the largest number of vaccinations. It should be noted that 52.0% of the population associated with this health department is not of Spanish origin [2]. In contrast, in the case of the pertussis vaccine, greater coverage was associated with older women and being of Spanish origin, coinciding with findings by other authors [2,18]. We can, therefore, deduce that the influenza vaccine has a lower degree of acceptance in the population born in Spain while that of pertussis is accepted by a larger portion of the population, coinciding with other studies [2,18]. Regarding parity, already having children was found to favour influenza vaccination, and having had at least one abortion constituted a rejection factor [36]. 

Coinciding with other studies, side effects reported by new mothers after vaccination were not serious, and they were reported to be mild for both vaccines [24,43]. No adverse effects were observed due to the joint inoculation of both vaccines [44]. 

### Strengths and Limitations

The strengths of the study include the fact that few studies have compared vaccination coverage recorded in registries to that declared by mothers. We were able to check that both data sources were in good agreement. Midwives correctly used the registry and pregnant women remembered the vaccinations they had received after the vaccination advice fairly well. Moreover, the sample was collected exhaustively, with a low rate of follow-up losses and a sample size that was large and sufficient for the estimations made. 

Limitations include the fact that telephone interviews were susceptible to possible interpretation errors to the extent that vaccinations mentioned by postpartum women may have been subject to recall bias. The survey is not exempted from a possible existence of interpretational errors or the inclusion of some information bias despite the simplicity of the items to be assessed. Although it is difficult to determine reasons for registry errors, whether due to excess (not reporting a vaccination but being registered as vaccinated in the NVR), or default (confirming to have been vaccinated though not being registered as vaccinated in the NVR), the error frequency was very low, which supported NVR estimates. To finish, we must remember that women who were not included in the NVR were not interviewed. 

## 5. Conclusions

The generalised availability of records such as the NVR used in this study would facilitate knowing pregnant women’s immunisation coverage, while also facilitating research on vaccine effectiveness and safety, which is essential for Public Health. The NVR is an effective platform for estimating immunisation coverage in pregnant women. The agreement between the declared vaccination and recorded vaccination was high for both vaccines: it was acceptable in the case of influenza vaccination and high for pertussis vaccination compared to other studies. Health professionals, especially midwives, play a fundamental role both in keeping records and recommending the vaccine to pregnant women. In addition to acting as facilitators of immunisation, they could be given a shared responsibility in the implementation of strategies to increase immunisation coverage.

Vaccinations in pregnant women should be analysed as an opportunity to improve coverage in all vaccines of the adult population that are low. The pregnancy situation or its planning is an opportunity for achieving a complete vaccination history of the woman. 

## Figures and Tables

**Table 1:** The frequencies n, percentages % of population distribution, according to study variables and data in the Nominal Vaccination Registry (NVR) *n* = 970.

Variables		Influenza Vaccinated *n* %	Influenza Unvaccinated *n* %	*p* *	Tdap Unvaccinated *n* %	Tdap Unvaccinated *n* %	*p* *	Total *n*
Vaccination coverage		623 64.2	347 35.8	NA	871 89.8	99 10.2	NA	970
Department	TV	297 73.7	106 26.3	0.001	347 86.1	56 13.9	0.001	403
	EC	326 57.5	241 42.5		524 92.4	43 7.6		567
Country of origin	Spain	421 60.8	271 39.2	0.001	635 91.8	57 8.2	0.001	692
	Not Spain	202 72.7	76 27.3		236 84.9	42 15.1		278
Previous pregnancies	0	21 60.0	14 40.0	0.003	31 88.6	4 11.4	0.917	35
	1	602 65.4	318 34.6		827 89.9	93 10.1		920
	≥2	7 33.3	8 66.7		13 83.3	2 16.7		15
Abortion	No	613 64.5	338 35.5	0.048	853 89.7	98 10.3	0.715	951
	Yes	10 60.0	9 40.0		18 93.3	1 6.7		19
Parity	0	18 43.9	23 56.1	0.019	37 90.2	4 9.8	0.176	41
	≥1	605 65.1	324 34.9		834 89.9	95 10.1		929
Visit to the midwife	Oct	141 71.9	55 28.1	0.031	-	-	NA	196
	Nov	423 63.3	245 36.7		-	-		668
	Dec	43 54.4	36 45.6		-	-		79
	Jan	16 59.3	11 40.7		-	-		27
Intention to be vaccinated	Yes	596 77.9	169 2 2.1	0.001	-	-	NA	765
	No	27 13.2	178 86.8		-	-		205
Vaccinated Tdap	Yes	560 57.7	311 32.0	0.52	-	-	NA	871
	No	63 6.5	36 3.7		-	-		99
Age	Mean SD	30.4 5.6	31.4 5.3	NA	31.0 5.3	29.1 6.6	NA	970

(*) *p*-values of Chi-square test to check the significance of the differences among categories. Tdap = Tetanus, Diphtheria, Pertussis for adults; NVR = Nominal Vaccination Registry; TV = Torrevieja; EC = Elche-Crevillente; Oct = October; Nov = November; Dec = December; Jan = January; SD = Standard Deviation; NA = No analysis.

**Table 2:** The pregnant women interviewed who also registered in the NVR (*n* = 663).

	Influenza				Tdap			
Women registered in NVR	vaccinated	unvaccinated	Total	CI (95%)	vaccinated	unvaccinated	Total	CI (95%)
	410 (61.8)	253 (38.2)	663 (100)		612 (92.3)	51 (7.7)	663 (100)	
Women interviewed	Were you vaccinated during the pregnancy?							
Yes	409 (61.7)	7 (1.1)	416 (62.7)	(58.1–67.3)	612 (92.3)	7 (1.1)	619 (93.4)	(91.4–95.3)
No	1 (0.2)	246 (37.1)	247 (37.3)	(31.3–43.3)	0 (0.0)	44 (6.6)	44 (6.6)	(0.0–13.9)

CI: Confidence Interval; Tdap = Tetanus, Diphtheria, Pertussis for adults.

**Table 3:** The side effects recorded for the influenza and pertussis vaccinations (*n* = 683).

**Influenza Vaccination**	***n* = 421/683 (61.6%)**		**CI (95%)**
Side effects	Yes	36/421 (8.6)	(0.0–17.7)
	No	385/421 (91.4)	(88.5–94.2)
Type of side effects	Premature birth	1/36 (2.7)	(0.0–8.0)
	Pain/Inflammation	12/36 (33.3)	(18.0–48.7)
	Flu	6/36 (16.7)	(4.5–28.8)
	Cold (with no fever)	17/36 (47.2)	(30.9–63.5)
**Pertussis Vaccination**	***n* = 630/683 (92.2%)**		**CI (95%)**
Side effects	Yes	67/630 (10.6)	(8.2–13.0)
	No	563/630 (91.4)	(89.2–93.6)
Type of side effects	Pain/Inflammation	61/67 (91.0)	(84.1–97.8)
	Fever at 24 h	6/67 (8.9)	(2.0–15.7)

CI = Confidence Interval.

## Abbreviations

VC	Valencian Community
ACs	Autonomous Communities
MSCBS	Ministerio de Sanidad, Consumo y Bienestar Social (Ministry of Health, Consumer Affairs and Social Welfare)
TV	Torrevieja
EC	Elx-Crevillent
AEMPS	Agencia Española del Medicamento y Productos Sanitarios (Spanish Agency of Medicines and Health Products)
NVR	Nominal Vaccination Registry.
